# Clinical impact of adherence to a standardized treatment algorithm for idiopathic sudden sensorineural hearing loss: a multicenter cohort study

**DOI:** 10.3389/fneur.2026.1775755

**Published:** 2026-02-18

**Authors:** Ryosuke Kitoh, Yutaka Takumi

**Affiliations:** Department of Otorhinolaryngology–Head and Neck Surgery, Shinshu University School of Medicine, Matsumoto, Japan

**Keywords:** guideline adherence, hearing recovery, idiopathic sudden sensorineural hearing loss, intratympanic corticosteroid, multicenter cohort, salvage therapy, systemic corticosteroid, treatment algorithm

## Abstract

**Background:**

Although systemic corticosteroids remain the mainstay of therapy for idiopathic sudden sensorineural hearing loss (SSNHL), evidence of adherence to standardized treatment algorithms and their impact on hearing outcomes is limited.

**Methods:**

We conducted a retrospective analysis of a prospectively collected multicenter cohort comprising one university hospital, 16 affiliated hospitals, and two community clinics in Nagano Prefecture, Japan (2018–2024). Consecutive patients diagnosed with SSNHL were treated according to a unified algorithm derived from national epidemiological surveys. Adherence was defined as compliance with the initial and salvage therapies specified in the algorithm, and hearing outcomes were evaluated using the five-frequency pure-tone average (250–4,000 Hz) according to national criteria. Multivariable logistic regression with robust standard errors was used to identify determinants of adherence and factors associated with marked recovery (≥30 dB improvement).

**Results:**

The overall adherence rate was 60.3% among 373 patients. Non-adherence was more prevalent in severe cases (Grade ≥3), among older patients (≥65 years), individuals with diabetes, and secondary referrals. Adherent cases showed better final hearing recovery than non-adherent ones (47.7% vs. 31.3%, *p* < 0.01). In cases of Grade ≥3, intravenous steroid therapy during initial treatment yielded greater early improvement than oral therapy (Grade 3: 46.0% vs. 25.0%, *p* = 0.018); however, there was no significant difference in final outcomes. Of the 110 patients requiring salvage treatment, intratympanic steroid injection was performed in 51.8% and was independently associated with superior recovery vs. observation (adjusted odds ratio, 14.35; 95% confidence interval, 1.60–128.42; *p* = 0.017).

**Conclusions:**

In this multicenter cohort, adherence to a standardized algorithm was moderate but was associated with improved hearing outcomes. In severe SSNHL, intravenous steroids enhance the early response—most notably in Grade 3—while salvage intratympanic steroid therapy considerably improves the final recovery rate. These findings support the implementation and optimization of the algorithm as well as the refinement of systemic dosing. Additionally, they highlight the importance of structured, guideline-based management in routine practice to improve outcomes in real-world SSNHL care.

## 1 Introduction

Idiopathic sudden sensorineural hearing loss (SSNHL) is the most common cause of acute unilateral hearing loss in adults and is frequently encountered in otolaryngology. Although several pathophysiological mechanisms have been hypothesized—including acute inflammation, circulatory disturbances, and autoimmune disease—it is difficult in routine practice to definitively assign a mechanism for each patient and tailor therapy accordingly.

**Figure 1:**
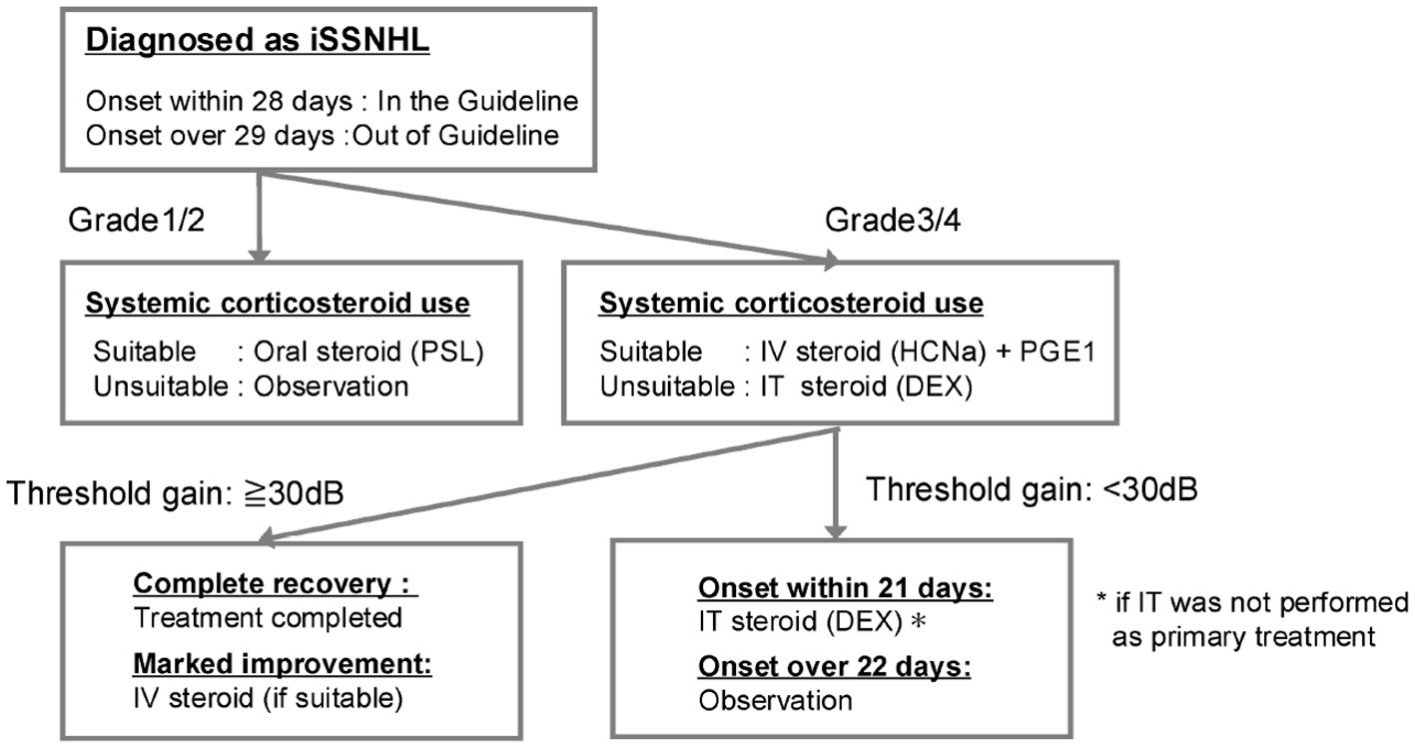
Treatment algorithm for idiopathic SSNHL based on a nationwide epidemiological survey in Japan (2014–2016). DEX, dexamethasone; HCNa, hydrocortisone sodium succinate; ISSNHL, idiopathic sudden sensorineural hearing loss; PGE_1_, prostaglandin E_1_; PSL, prednisolone.

Corticosteroid therapy is the usual focus of treatment discussions; however, its efficacy has not been definitively established, and both domestic and international guidelines offer only limited strong recommendations for specific treatments (1–4). In Japan, periodic nationwide epidemiologic surveys have been conducted by the Ministry of Health, Labour and Welfare research groups on acute sensorineural hearing loss and, later, the Research Group to treat intractable hearing diseases. In the 2014–2016 survey, approximately 3,400 cases were collected (5). The treatment content was analyzed, revealing that steroids were administered in 92% of the patients. Although the evidence level for systemic steroids in SSNHL is not high, this background informed the development of a practical, epidemiology-based treatment algorithm for clinical use, which we have previously reported (Figure 1) (6).

The American Academy of Otolaryngology–Head and Neck Surgery (AAO-HNS) guideline also presents a treatment algorithm (3). However, a study examining adherence to this guideline reported that systemic steroids were administered in only 54.5% of the cases and intratympanic (IT) steroids in 18.5%, suggesting that a substantial proportion of cases deviated from the algorithm (7).

We aimed to quantify the adherence to a standardized SSNHL treatment algorithm across a multicenter network, identify the clinical determinants of non-adherence, and examine whether adherence is associated with improved hearing recovery. We hypothesized that higher adherence would be associated with better outcomes.

## 2 Materials and methods

### 2.1 Study design

This multicenter, prospective cohort study was conducted in Nagano Prefecture, Japan, and data were analyzed retrospectively. The participating sites included Shinshu University Hospital, 16 affiliated hospitals, and two community clinics (see Supplementary Table S1 for a list of the participating facilities). Although adherence to the algorithm reported in 2019 was the basic policy, alternative treatment was permitted when a patient refused or when the attending physician deemed the algorithmic therapy infeasible. Adherence was defined as compliance with both initial and salvage therapies specified in the algorithm. Cases that did not follow the algorithm were considered non-adherent (*i.e*., deviated from the algorithm).

The diagnostic criteria for SSNHL, severity grading used in the algorithm, and outcome criteria were defined by the Japanese Ministry of Health, Labour and Welfare (Supplementary Table S2) (1, 2).

### 2.2 Participants and procedures

A total of 373 consecutive patients with SSNHL who visited participating facilities between 2018 and March 2024 were included in this study. We excluded cases with obvious external causes (*e.g*., trauma), known viral diseases that cause hearing loss, or central nervous system disorders. First, to assess overall adherence, we determined whether each patient followed or deviated from the algorithm. We also analyzed the factors associated with adherence.

As deviations were frequent among patients with an initial average hearing level of ≥60 dB (Grade ≥3), we compared hearing improvement between adherent and non-adherent care for two specific, common non-adherent patterns in this subgroup: (1) choosing oral instead of intravenous (IV) systemic steroid therapy as the initial treatment, and (2) not performing IT steroid injection as salvage treatment in those who needed salvage. The “average hearing level” and thresholds refer to the five-frequency pure-tone averages (250, 500, 1,000, 2,000, and 4,000 Hz) used in Japan's grading and outcome criteria.

For the initial treatment of Grade ≥3 cases in the algorithm, IV steroids are combined with a prostaglandin E_1_ (PGE_1_) preparation. This specification was based on the results of a nationwide epidemiological survey conducted by the Ministry of Health, Labour and Welfare, in which combination therapy with systemic steroids and PGE_1_ was associated with a significantly better hearing prognosis than steroids alone (8). In the present study, we defined deviation as the use of oral steroids in these severe cases. For IV steroids, a tapering dose of 300 mg/day hydrocortisone sodium succinate was administered. No dose was prescribed for severe cases that were managed with oral therapy. Prednisolone (30 mg/day) was tapered for Grade 1/2 oral therapy.

The definition of the need for salvage treatment was ≤ 30 dB improvement in average hearing at completion of initial therapy. The hearing test closest to and within 1 week after the end of the initial therapy was used to judge early response. Our algorithm recommends IT steroid injection within 21 days of onset as salvage therapy, informed by prior institutional data showing better hearing improvement with ≤ 21-day initiation (9). Cases in which this was not performed were considered non-adherent (*i.e*., salvage therapy was needed, but either no salvage was administered or a non-IT alternative was used).

IT dexamethasone administration was primarily performed as transtympanic dexamethasone injection. Local anesthesia was achieved by iontophoresis for the first session and by topical anesthesia of the tympanic membrane using a local anesthetic solution for subsequent sessions. A 23- or 25-gauge needle was used to slowly inject approximately 0.3–0.8 ml of dexamethasone (3.3 mg/ml) into the middle ear. After injection, patients were instructed to remain for 30 min with the treated ear upward, either in the lateral decubitus position (affected ear up) or in the supine position with the jaw elevated and the head turned approximately 45° toward the contralateral side. Injections were performed for a total of four sessions, typically on alternate days.

### 2.3 Outcomes

For the final (fixed) hearing outcome, effectiveness was defined as “marked improvement or better,” *i.e*., ≥30 dB threshold improvement according to national criteria (Supplementary Table S2). The fixed outcome was assessed at least 2 months after onset. We also compared absolute threshold changes between the groups. Four patients lacked a fixed-time audiogram and were excluded from the final outcome analyses.

### 2.4 Statistical analysis

Statistical analyses were performed using IBM SPSS Statistics for Windows, Version 28.0 (IBM Corp., Armonk, NY, USA). Group comparisons were performed using the chi-square test or Fisher's exact test, as appropriate. Logistic regression was used to analyze determinants of adherence and factors associated with outcomes. Results are reported as adjusted odds ratios (aORs) with 95% confidence intervals (CIs). Robust standard errors were applied, and facility type was included as a categorical covariate. Model discrimination was assessed using the area under the curve (AUC), and multicollinearity was evaluated using the variance inflation factor (VIF). A two-sided *p* < 0.05 was considered statistically significant. Complete-case analysis was also performed. Although no *a priori* sample size calculation was performed, given the sample size (*n* = 373) and the prespecified set of covariates, the study was considered adequately powered to detect moderate effect sizes in logistic regression models with a two-sided α = 0.05.

The covariates were prespecified *a priori* based on prior SSNHL literature and clinical plausibility. These included age (≥65 years), vertigo, diabetes, initial severity grade, days from onset to treatment, treatment status (primary vs. secondary), and facility.

Missingness patterns were inspected. Due to the low-to-moderate item-level missingness and plausibility of missing data at random, complete-case analyses were used as the primary approach.

During manuscript preparation, we used ChatGPT (OpenAI; model GPT-5.2 Pro; web interface; accessed December 2025) to assist with English-language editing and to review the clarity and consistency of the statistical analysis description. The tool was not used to analyze data or generate results; the authors take full responsibility for the content.

### 2.5 Ethical considerations

This study adhered to the principles of the Declaration of Helsinki. The protocol was approved by the Ethics Committee for Life Science and Medical Research at Shinshu University, as well as by each participating facility (Shinshu University approval No. 6241).

## 3 Results

### 3.1 Cohort characteristics

Table 1 summarizes the 373 cases. Some symptom and comorbidity variables had item-level missingness; therefore, percentages were computed after excluding missing values for each item. The facilities included clinics for 74 (19.8%), affiliated hospitals for 202 (54.2%), and the university hospital for 97 (26.0%) patients. Initial treatment was started within the study network for 311 patients (83.4%), while 56 (15%) were secondary referrals after receiving initial treatment elsewhere, and 6 (1.6%) received no treatment. The mean initial affected-ear pure-tone average (PTA) was 59.5 dB (SD 26.1). The severity grades were as follows: Grade 1, *n* = 102; Grade 2, *n* = 109; Grade 3, *n* = 99; Grade 4, *n* = 63. Systemic corticosteroids were used in 346 patients (92.8%), PGE_1_ was combined in 90 (24.1%), and IT steroid injection was performed in 72 (19.3%).

**Table 1:** Characteristics of the included patients.

**Characteristic**	**Value**	**Percentage (%)**
**Number of patients with SSNHL**	373	
**Sex**		
Male: Female	186: 187	
**Age of onset**		
Average (SD)	60.5 (SD: 16.8)	
Median	63	
**Facility**		
Clinic	74	19.8
Affiliated hospital	202	54.2
University hospital	97	26.0
**Treatment status**		
Primary	311	83.4
Secondary referral	56	15.0
No treatment	6	1.6
**Time from the onset to the start of treatment**		
Average (SD)	5.0 (SD: 9.3)	
Median	3	
**Pre-treatment hearing level of affected side**		
Average (SD)	59.5 (SD:26.1)	
Median	54	
**Pre-treatment hearing level of unaffected side**		
Average (SD)	23.1 (SD: 15.4)	
Median	20	
**Grade of hearing loss**		
1:2:3:4	102:109:99:63	
**Symptom**		
Tinnitus	204	67.1
Ear fullness	152	71.7
Vertigo/dizziness	122	34.8
**Comorbidity**		
Diabetes mellitus	63	18.8
Hypertension	103	31.5
Hyperlipidemia	57	18.0
**Treatment**		
Systemic corticosteroid	346	92.8
Prostaglandin E1	90	24.1
Intratympanic steroid	72	19.3

### 3.2 Non-adherence to the algorithm

Overall, adherence was observed in 225 of the 373 cases (60.3%), while non-adherence was observed in 148 (39.7%). In the univariate analyses (chi-square), non-adherence was considerably more prevalent among patients aged ≥65 years, those treated at hospital settings (affiliated or university), those receiving secondary treatment, those with greater initial severity (especially Grade ≥3), and those with diabetes (Table 2).

**Table 2:** Univariate analyses of factors related to adherence.

**Variable**	**Adherent**	**Non-adherent**	**Adherence rate (%)**	***p*-value**
**Sex**				
Male	105	81	56.5	0.139
Female	120	67	64.2	
**Age**				
< 65	144	55	72.4	**< 0.001**
≥65	81	93	46.6	
**Facility**				
Clinic	60	14	81.1	**< 0.001**
Affiliated hospital	115	87	56.9	
University hospital	50	47	51.5	
**Treatment status**				
Primary	209	102	67.2	**< 0.001**
Secondary referral	15	41	26.8	
**Time from the onset to the start of treatment**				
≤ 7	187	124	60.1	0.888
≥8	38	24	61.3	
**Grade of hearing loss**				
Grade 1/2	170	41	80.6	**< 0.001**
Grade 3/4	55	107	34.0	
**Comorbidity**				
**Diabetes mellitus**				
Positive	29	34	46.0	**0.022**
Negative	171	102	62.6	
**Hypertension**				
Positive	55	48	53.4	0.147
Negative	139	85	62.1	
**Hyperlipidemia**				
Positive	28	29	49.1	0.104
Negative	158	101	61.0	

In univariate tests, older age (≥65 years), hospital setting (university), secondary treatment, higher initial severity (Grade 3/4), and diabetes were associated with non-adherence to the algorithm. Bold *p*-values indicate statistical significance.

Because univariate comparisons may be confounded by disease severity, referral status, and institutional case-mix, we modeled adherence (yes/no) using multivariable logistic regression and report aORs with 95% CIs. The covariates included age, severity, treatment status (primary vs. secondary), facility type (clinic [reference] vs. affiliated vs. university), comorbidities (diabetes, hypertension, dyslipidemia), and days from onset to start of initial treatment (0–3 [reference], 4–7, 8–14, and ≥15). Robust standard errors were used. Severe hearing loss (aOR 0.08), secondary treatment (aOR 0.04), age ≥65 (aOR 0.38), and diabetes (aOR 0.31) were associated with lower adherence. While unadjusted analyses suggested lower adherence at the university hospital, after adjusting for the concentration of secondary referrals, the university setting showed higher adherence than the clinics (aOR 9.59). Model discrimination was good (AUC 0.86), and multicollinearity was not a concern (max VIF, 3.56; Table 3).

**Table 3:** Multivariable analysis of factors related to adherence (logistic regression).

**Variable**	**aOR (95% CI)**	***p*-value**
Facility (Affiliated hospitals)	0.529 (0.246–1.138)	0.103
Facility (University hospital)	9.593 (2.935–31.357)	**<0.001**
Age (≥65)	0.38 (0.193–0.748)	**0.005**
Grade of hearing loss (≥3)	0.08 (0.038–0.169)	**<0.001**
Treatment status (secondary referral)	0.04 (0.012–0.139)	**<0.001**
Diabetes mellitus	0.305 (0.129–0.725)	**0.007**
Hypertension	2.108 (1.019–4.363)	**0.044**
Hyperlipidemia	1.272 (0.523–3.095)	0.595
Treatment start (days 4–7)	1.341 (0.597–3.014)	0.478
Treatment start (days 8–14)	1.099 (0.424–2.848)	0.847
Treatment start (days ≥15)	1.467 (0.291–7.401)	0.642

aOR, adjusted odds ratio; CI, confidence interval. Bold *p*-values indicate statistical significance.

Among the 162 patients with Grade ≥3, the most frequent non-adherent patterns (not mutually exclusive) were oral steroids for initial treatment (*n* = 63) and no IT steroids despite salvage indication (*n* = 53). In contrast, the most common non-adherent action in Grade ≤ 2 was the omission of any systemic corticosteroid at initial treatment (*n* = 17). In 16 other Grade ≤ 2 cases, inpatient IV steroids were administered (more intensive than the algorithm), and in two Grade 1 cases, IT steroids were administered as salvage treatment due to strong patient request. Accordingly, in Grade ≤ 2, both under- and over-intensification occurred.

### 3.3 Adherence vs. treatment outcomes

**Figure 2:**
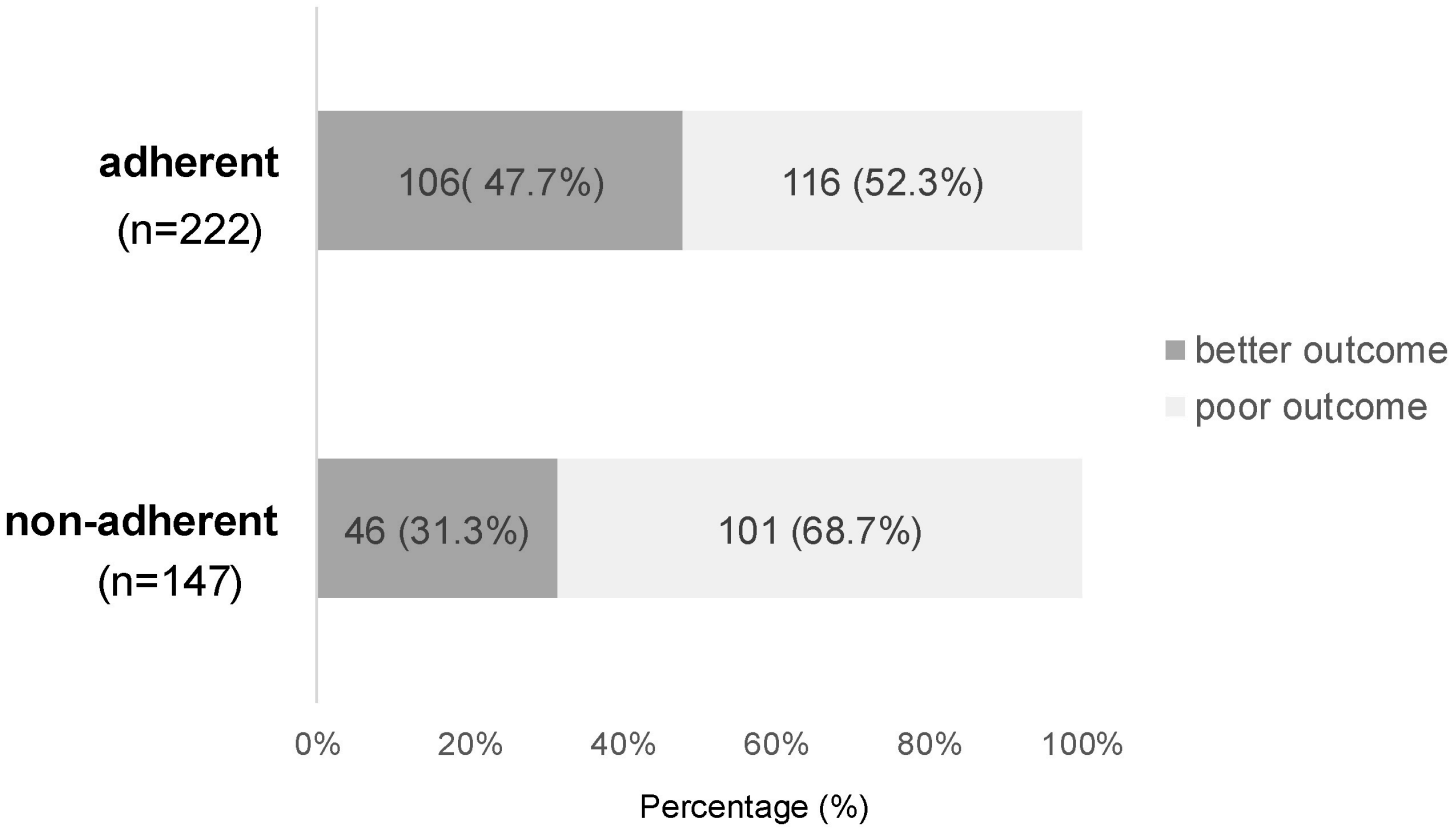
Relationship between algorithm adherence/non-adherence and outcome category. “Better outcome” indicates cases classified as complete recovery or marked improvement by the national criteria; “poor outcome” indicates slight improvement or no change. Better outcomes were observed in 47.7% of adherent cases and 31.3% of non-adherent cases (*p* = 0.002, Fisher's exact test).

Figure 2 shows the correlation between adherence and the final outcomes. “Marked improvement or better” (≥30 dB improvement) occurred in 47.7% (106/222) of adherent cases vs. 31.3% (46/147) of non-adherent cases (*p* = 0.002, Fisher's exact test).

We subsequently focused on Grade ≥3 cases and examined two common non-adherent patterns: (1) initial route of systemic steroids (oral vs. IV) and (2) salvage IT steroid injections (performed vs. not performed).

#### 3.3.1 Effect of initial steroid route in severe cases

Among Grade ≥3 cases, we excluded nine patients who did not initially receive systemic corticosteroids and one patient whose final outcome was unknown. This left 152 patients: 90 were treated with IV steroids according to the algorithm, and 62 received oral steroids, deviating from this treatment.

We assessed efficacy at two time points: (1) at the completion of the initial therapy (early response) and (2) at fixed hearing (final outcome).

**Figure 3:**
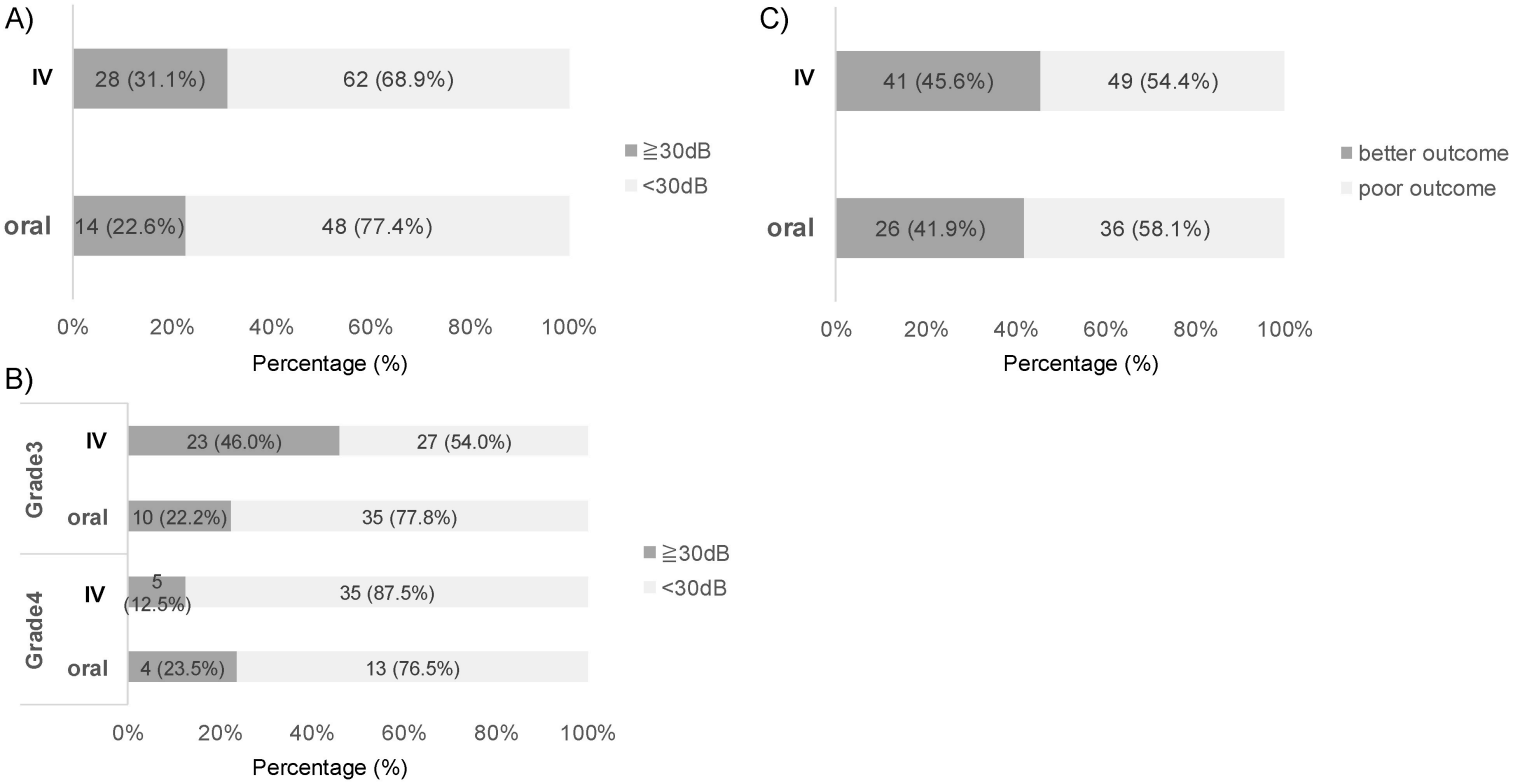
Comparison of IV vs. oral systemic steroids as initial therapy in severe cases. **(A)** Early response at completion of initial therapy: ≥30 dB improvement in 31.1% with IV and 22.6% with oral; difference not significant (*p* = 0.273, Fisher's exact test). **(B)** Grade-stratified early response: Grade 3 showed significantly more ≥30 dB improvements with IV than oral (*p* = 0.018, Fisher's exact test), whereas Grade 4 showed no significant difference (*p* = 0.428). **(C)** Final (fixed) outcomes: no significant difference between IV and oral groups (*p* = 0.740).

*Early response*: an improvement of ≥30 dB occurred in 31.1% (28/90) of the IV group and 22.5% (14/62) of the oral group (Figure 3A), which was not statistically significant overall. However, when stratified by grade, Grade 3 cases showed a significantly higher proportion of early responders with IV therapy (46.0% [23/50]) than with oral therapy (25.0% [11/44], *p* = 0.018). In contrast, Grade 4 cases showed low response rates in both groups, with no significant differences (Figure 3B).

*Final outcome*: At fixed hearing, “marked improvement or better” was observed in 45.6% (41/90) of the IV group and 41.9% (26/62) of the oral group (Figure 3C). The mean threshold improvement from baseline was 26.9 dB (SD 21.9) vs. 23.9 dB (SD 21.7), respectively, but neither difference was significant. Grade-stratified analyses showed no significant differences between the groups.

#### 3.3.2 Effect of IT steroid salvage

**Figure 4:**
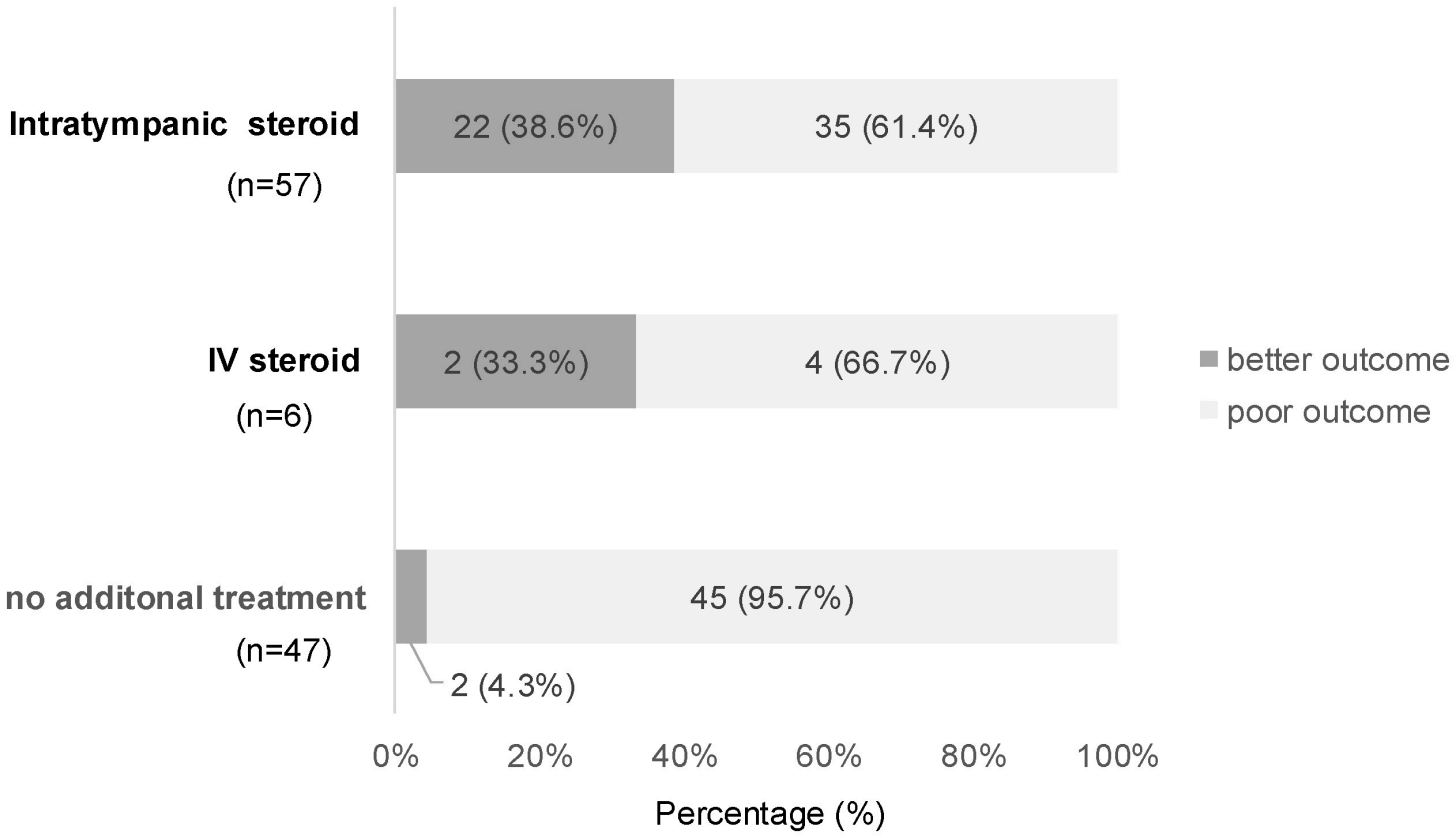
Final outcomes in salvage-eligible cases by salvage modality. Using fixed-time outcome criteria, the proportions with “better outcome” were 38.6% for IT steroids, 33.3% for IV steroids, and 4.3% for no additional treatment, with a significant overall difference (*p* < 0.001, Fisher's exact test).

Of the 152 severe cases, 110 failed to achieve ≥30 dB improvement by completion of initial therapy and thus met the criteria of salvage treatment. Among them, 57 (51.8%) received IT steroid injections in accordance with the algorithm, six (5.5%) received IV steroids as an alternative, and 47 (42.7%) received no additional treatment. The final outcomes (Figure 4) showed “marked improvement or better” in 38.6% (22/57) of the IT group, 33.3% (2/6) of the IV group, and 4.3% (2/47) of the observation group (*p* < 0.001).

A multivariable logistic regression model (complete cases *n* = 108) was used to assess factors associated with achieving final “marked improvement or better” among salvage-eligible patients (Table 4). The covariates included IT steroids (yes/no), days to the start of IT (0–7 [ref], 8–14, 15–21; modeled as an interaction with IT, since non-IT cases lack a “start day”), Grade 4 vs. 3, vertigo, age ≥65, and secondary vs. primary treatment. IT steroids were independently associated with better outcomes [aOR 14.35, 95% CI 1.60–128.42; *p* = 0.017; average marginal effect (AME) +33.1%, 95% CI +5.3 to +58.6]. Vertigo (aOR 0.22; *p* = 0.031; AME −18.3%, 95% CI −32.6 to −1.6) and age ≥65 (aOR 0.15; *p* = 0.003) were unfavorable. Grade 4 (vs. Grade 3) tended to have worse outcomes, but the difference was not statistically significant (aOR 0.73; *p* = 0.636). The interaction terms for IT timing (8–14 days and 15–21 days vs. 0–7 days) were directionally unfavorable but not significant. Model discrimination was good (AUC 0.879).

**Table 4:** Multivariable analysis of factors affecting final outcome among salvage-eligible patients.

**Variable**	**aOR (95% CI)**	***p*-value**
IT steroid	14.354 (1.604–128.424)	**0.017**
IT × start day (days 8–14)	0.454 (0.059–3.500)	0.448
IT × start day (days 15–21)	0.303 (0.031–2.971)	0.305
Grade 4 (vs. 3)	0.732 (0.201–2.664)	0.636
Vertigo (present)	0.220 (0.056–0.872)	**0.031**
Age (≥65)	0.152 (0.044–0.524)	**0.003**
Treatment status (secondary referral)	0.198 (0.035–1.114)	0.066

IT steroids were positively associated with “marked recovery or better,” whereas vertigo and age ≥65 years were associated with poorer outcomes.

aOR, adjusted odds ratio; CI, confidence interval; IT, intratympanic. Bold *p*-values indicate statistical significance.

## 4 Discussion

This multicenter analysis demonstrated that adherence to a standardized SSNHL treatment algorithm in the real world is associated with better hearing recovery. This finding reinforces the value of structured care pathways in routine practice.

From an implementation perspective, non-adherence was found to cluster among older patients, those with diabetes, and secondary referrals. Providing targeted education to referral providers, creating streamlined inpatient pathways to ensure timely initiation of appropriately dosed systemic corticosteroids, and proactive scheduling of salvage IT steroid injection within 21 days could improve adherence and outcomes without altering the algorithm. These findings provide practical insights into quality improvement initiatives, particularly in settings where coordination between primary and secondary care can delay the timely initiation of appropriately dosed systemic steroids and the scheduling of salvage IT steroid injection within 21 days.

We applied a standardized, epidemiology- and evidence-informed treatment algorithm for SSNHL across diverse clinical settings and assessed adherence and its consequences. The overall adherence rate was approximately 60%, with higher non-adherence rates among Grade ≥3 patients, those aged ≥65 years, secondary referrals, and patients with diabetes. Common non-adherent patterns included the use of oral rather than IV systemic corticosteroids in severe cases; this deviation was associated with a poorer early response in Grade 3 patients. Conversely, omitting IT steroids as a salvage treatment was associated with poorer final outcomes.

*Adherence to treatment algorithms*: Previous research by the AAO-HNS has examined adherence to 16 guidelines since 2004, revealing an average adherence rate of 56%. For SSNHL-related items, adherence among subspecialists was generally high, except for IT steroid administration (7, 10, 11). A CHEER Network study found that only 63.6% of 173 patients received systemic or IT steroids.

In our framework, the algorithm specifies oral therapy for Grade 1/2 and IV therapy for Grade 3/4. Therefore, the 63 severe cases treated orally were considered non-adherent. However, if “adherence” were defined more broadly to include any systemic or IT steroids for initial therapy, 94.4% (352/373) of the patients would be considered adherent, which is higher than that reported in the CHEER study. Among those with a poor early response, 51.8% received IT steroids as salvage therapy, which is higher than that in previous US reports. This suggests that our algorithm aligns well with real-world Japanese practice.

Initial steroid route and treatment effect: The algorithmic preference for oral steroids (Grade 1/2) vs. IV steroids (Grade 3/4) reflects the frequencies of treatment observed in the 2014–2016 epidemiology survey. In our study, 41.4% (63/152) of the severe cases receiving systemic steroids were treated orally. The only significant predictor of choosing oral therapy was the secondary treatment status. Age, sex, initial grade, vertigo, and comorbidities showed no significant associations. At the university hospital, only 6.7% (2/30) of the primary cases were treated orally; of those for which the reasons were recorded, approximately 80% were due to prior oral treatment started at the referring facility. In affiliated hospitals and clinics, where there were fewer secondary referrals, most oral therapy non-adherence was likely due to the refusal of inpatient care (this detail was not always recorded). Specific reasons for choosing outpatient oral therapy were not systematically captured; however, where documented, two recurring contexts were identified: (i) secondary referrals who had already started oral steroids at the referring facility and (ii) primary cases managed orally because admission for IV therapy was declined or impractical. Less commonly, practical constraints such as difficulty securing IV access were noted.

The AAO-HNS lists prednisolone, methylprednisolone, and dexamethasone as initial therapy options without mandating the IV or oral route. The suggested prednisolone dose is approximately 1 mg/kg/day, capped at 60 mg/day (3). The optimal dose has been debated; some retrospective studies favored higher doses (12, 13), whereas others (including a recent randomized controlled trial comparing high-dose intravenous prednisolone/dexamethasone vs. tapering from 60 mg) found no superiority of the high dose (14, 15). In our cohort, the intravenous therapy commonly used was hydrocortisone at a dose of 300 mg/day (equivalent to 75 mg prednisolone in terms of anti-inflammatory activity), which is close to 1 mg/kg/day for many adults. In contrast, oral therapy often defaulted to a dose of 30 mg prednisolone per day (in accordance with the Grade 1/2 protocol), which is lower than the typical recommendation for severe hearing loss. This dosing disparity likely contributed to the better early response observed with IV therapy in Grade 3 patients. However, the final outcomes did not differ significantly after subsequent care. Importantly, we observed little within-route variation in systemic dosing in this cohort (most oral regimens used prednisolone 30 mg/day and most IV regimens used hydrocortisone 300 mg/day), which limited formal dose-response analyses. Therefore, the observed early benefit of the algorithmic IV regimen in Grade 3 should be interpreted as reflecting the difference in overall glucocorticoid exposure between the commonly used regimens rather than definitive evidence that the IV route itself is superior. Future studies that capture weight-based dosing and detailed taper schedules are needed to refine the optimal systemic regimen, including whether standardized higher-dose oral regimens could mitigate potential underdosing when admission is not feasible.

Salvage IT steroids: For Grade ≥3 cases with a poor early response (less than 30 dB improvement), the algorithm recommends IT steroids within 21 days. In practice, 53 of the 110 eligible patients did not receive IT steroids, primarily because IT was offered but declined. Nevertheless, IT steroids produced considerably better final outcomes than observation alone, and multivariable modeling confirmed their independent association with “marked recovery or better.” This supports the proactive recommendation of IT steroids during salvage therapy, regardless of the initial systemic route or dose. In the few cases with recorded reasons for omitting IT salvage, the most common reason was patient refusal; rare practical or anatomic factors (e.g., prior tympanic surgery) were also noted. These patterns suggest that standardized counseling and rapid-access scheduling may be key to improving real-world uptake of guideline-recommended salvage IT therapy.

In recent years, multiple systematic reviews and meta-analyses have demonstrated the efficacy of IT steroid injection as a salvage therapy (16–19). These syntheses consistently demonstrate that IT steroids are more effective than a placebo or no additional treatment. Furthermore, several studies support IT corticosteroid injection as an effective salvage option for refractory SSNHL (20). Consistent with this evidence, the AAO-HNS guideline recommends salvage IT steroid injection (3).

Although the combination of systemic and IT therapies has been reported to be beneficial in some studies, IT injections carry the risk of persistent tympanic membrane perforation. Our previous data indicated an incidence rate of approximately 8.7%, while a systematic review reported a rate of around 1% (with a range of up to 20%) (21, 22). As some patients recover adequately with systemic therapy alone, we currently reserve IT for salvage rather than as a routine upfront combination.

*Limitations and originality*: This was not a randomized trial; therefore, causal inferences regarding specific regimens were limited. The inclusion of secondary referrals initially treated outside the network makes the precise estimation of adherence and efficacy more difficult. Among the primary cases treated within the network, adherence was 67.2% (209/311), which was higher than the overall rate of 60.3%. It is also difficult to quantify the total number of patients who completed the initial therapy at non-participating clinics, and their outcomes. To our knowledge, this is the first multicenter study in Japan to quantify real-world adherence to a standardized SSNHL algorithm and demonstrate its independent association with hearing recovery across a network of university hospitals, affiliated hospitals, and clinics.

The strength of this study lies in its unified initial treatment policy and prospective case capture across diverse facilities, which allows for a practical assessment of which aspects are likely to deviate and the flexibility an algorithm can tolerate. Our findings particularly support the recommendation of salvage IT steroid injections for severe cases with a poor early response, independent of the initial systemic route or dose. Even with good algorithm adherence, “marked improvement or better” was achieved in 47.7% of the overall cases and 63.6% of the severe cases in the specified subset analyses, leaving room for improvement. Priorities include: (1) broader dissemination and implementation support for adherence; (2) reconsideration of oral dosing (especially in severe cases initially treated outside hospitals); and (3) development of novel therapies for groups with persistently poorer outcomes (Grade 4, age ≥65, and vertigo).

## 5 Conclusions

In this multicenter study spanning a university hospital, affiliated hospitals, and clinics, we evaluated adherence to an SSNHL treatment algorithm and the impact of common deviations. Overall adherence was found to be 60.3%. Deviations were more prevalent in severe cases, secondary referrals, patients aged ≥65 years, and those with diabetes. Patients who adhered to the treatment protocol showed better outcomes than those who did not. Upon comparing IV vs. oral systemic steroids as initial therapy in severe cases, only early response in Grade 3 favored IV; the final outcomes did not differ significantly. IT steroid injection was performed in approximately 52% of salvage-eligible patients and was associated with markedly better final outcomes than observation alone.

To improve results, we propose the following: (1) implementing strategies to enhance adherence; (2) revisiting oral steroid dosing where underdosing is likely; and (3) exploring new treatments for persistently poor-prognosis groups (Grade 4, age ≥65, and vertigo).

## Data Availability

The raw data supporting the conclusions of this article will be made available by the authors, without undue reservation.

## Supplementary material

Supplementary Figure 1 Patient flow for cohort enrollment, analysis sets, and salvage eligibility. This diagram summarizes the study flow from all enrolled patients with idiopathic SSNHL (*n* = 373) to analytic subsets. The severe subgroup (Grade ≥3) used for the comparison of initial systemic corticosteroid route comprised 152 patients after excluding those who did not receive initial systemic corticosteroids (*n* = 9) and those who did not undergo a final hearing assessment (*n* = 1). Among severe cases who did not achieve ≥30 dB improvement by completion of initial therapy (salvage-eligible, *n* = 110), salvage modalities are shown: IT steroid injection (*n* = 57), IV steroids (*n* = 6), or observation with no additional treatment (*n* = 47). Subset labels indicate the corresponding main-text sections: overall adherence (Section 3.2), impact of initial steroid route in severe cases (Section 3.3.1), and effect of IT steroid salvage (Section 3.3.2). Severity grading and outcome definitions followed the national criteria used in Japan (see Supplementary Table S2). IT, intratympanic; IV, intravenous; PTA, pure-tone average; SSNHL, sudden sensorineural hearing loss.

Supplementary Figure 2 Determinants of adherence to the standardized SSNHL treatment algorithm (multivariable logistic regression; forest plot). The forest plot displays the aORs with 95% CIs for adherence (yes = 1) vs. non-adherence (no = 0) to the algorithm. The covariates encompassed age ≥65 years, initial severity grade, treatment status (primary vs. secondary), facility type (clinic [reference], affiliated hospital, university hospital), comorbidities (diabetes, hypertension, dyslipidemia), and days from onset to start of initial therapy (0–3 [reference], 4–7, 8–14, ≥15). Estimates were obtained using logistic regression with robust standard errors. Model discrimination: AUC = 0.86; maximum VIF = 3.56. Values >1 indicate higher odds of adherence; values < 1 indicate lower odds of adherence. Full numerical results are provided in Table 3 of the main text. aOR, adjusted odds ratio; AUC, area under the curve; CI, confidence interval; VIF, variance inflation factor.

Supplementary Figure 3 Determinants of “marked improvement or better” among salvage-eligible severe SSNHL cases (multivariable logistic regression; forest plot). Among Grade ≥3/4 patients who were salvage-eligible after initial therapy ( ≤ 30 dB improvement at completion of initial therapy), this forest plot shows aORs with 95% CIs for achieving “marked improvement or better” (≥30 dB improvement) at the fixed hearing assessment. The covariates included IT steroids (yes vs. no), timing of IT (0–7 days [reference], 8–14, 15–21 days from onset; modeled as an interaction with IT), initial severity (Grade 4 vs. 3), vertigo (present vs. absent), age ≥65 years, and treatment status (secondary vs. primary). Logistic models used robust standard errors; model AUC = 0.879. In the main text (Table 4), IT steroids were independently associated with better outcomes (aOR 14.35; 95% CI 1.60–128.42; *p* = 0.017), whereas vertigo and age ≥65 years were associated with poorer outcomes. aOR, adjusted odds ratio; AUC, area under the curve; CI, confidence interval; IT, intratympanic; SSNHL, sudden sensorineural hearing loss.

Table S1 List of participating facilities in the multicenter study.

Table S2 SSNHL diagnostic criteria, severity grading, and outcome criteria used in Japan: (A) diagnostic criteria for idiopathic SSNHL; (B) severity grading criteria; (C) hearing improvement criteria.
