# Supplementary figures and images for: Clinical impact of adherence to a standardized treatment algorithm for idiopathic sudden sensorineural hearing loss: a multicenter cohort study
Source: Front Neurol. 2026 Feb 18;17:1775755. doi: 10.3389/fneur.2026.1775755 (PMC12956656; doi:10.3389/fneur.2026.1775755)

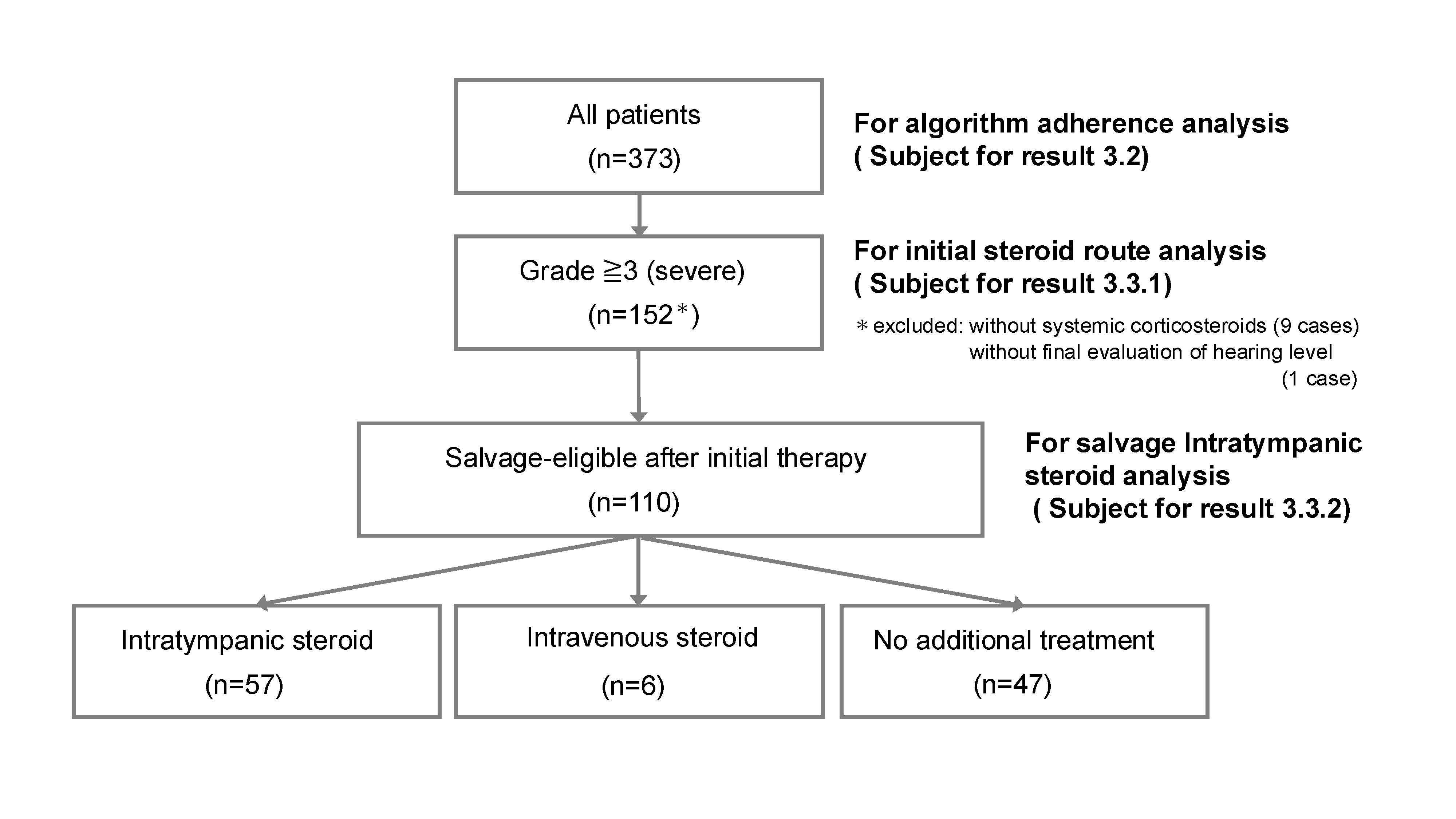

Supplement: Supplementary Figure 1 — Patient flow for cohort enrollment, analysis sets, and salvage eligibility. This diagram summarizes the study flow from all enrolled patients with idiopathic SSNHL (n = 373) to analytic subsets. The severe subgroup (Grade ≥3) used for the comparison of initial systemic corticosteroid route comprised 152 patients after excluding those who did not receive initial systemic corticosteroids (n = 9) and those who did not undergo a final hearing assessment (n = 1). Among severe cases who did not achieve ≥30 dB improvement by completion of initial therapy (salvage-eligible, n = 110), salvage modalities are shown: IT steroid injection (n = 57), IV steroids (n = 6), or observation with no additional treatment (n = 47). Subset labels indicate the corresponding main-text sections: overall adherence (Section 3.2), impact of initial steroid route in severe cases (Section 3.3.1), and effect of IT steroid salvage (Section 3.3.2). Severity grading and outcome definitions followed the national criteria used in Japan (see Supplementary Table S2). IT, intratympanic; IV, intravenous; PTA, pure-tone average; SSNHL, sudden sensorineural hearing loss. [file Image_1.tiff]

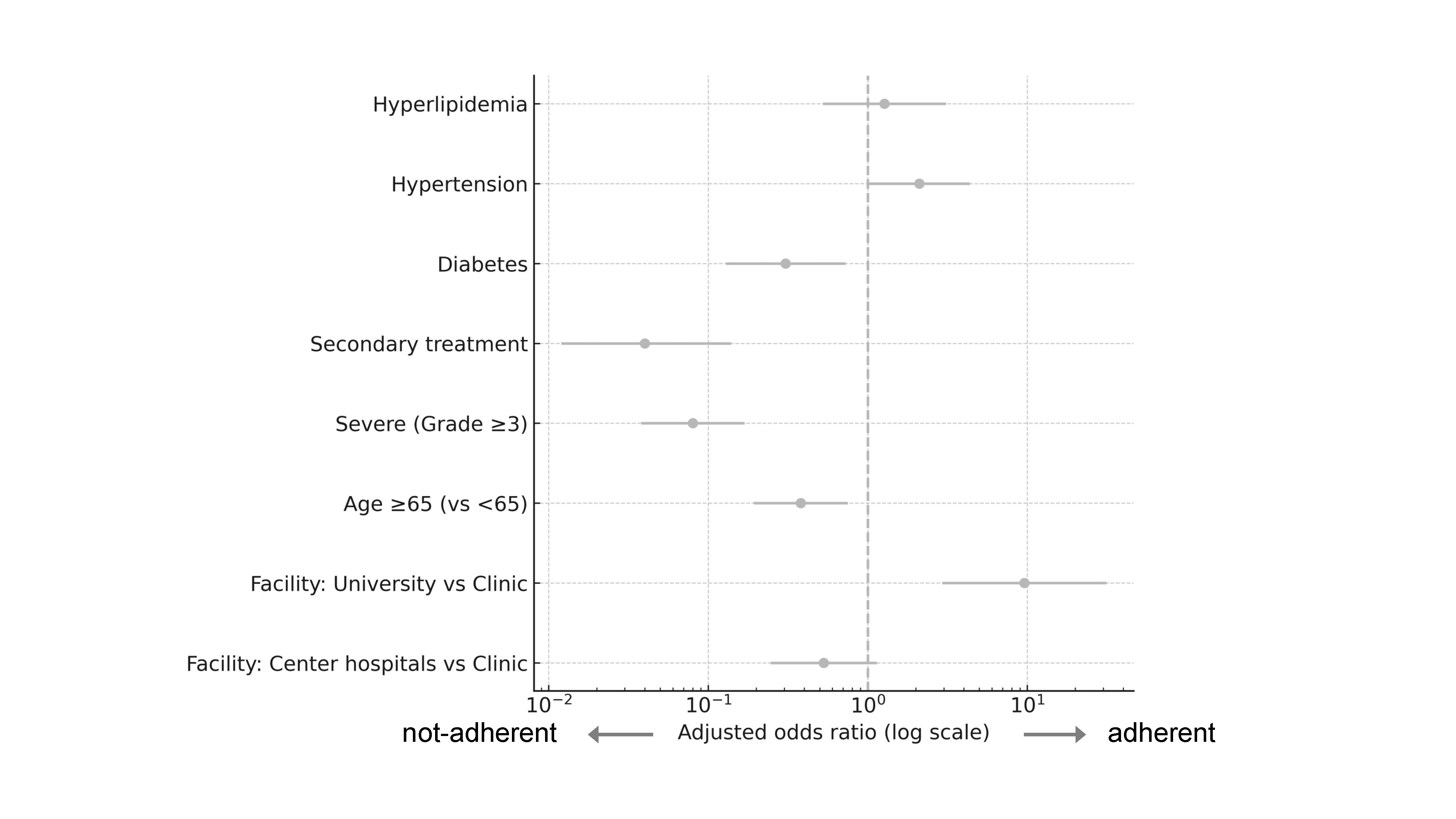

Supplement: Supplementary Figure 2 — Determinants of adherence to the standardized SSNHL treatment algorithm (multivariable logistic regression; forest plot). The forest plot displays the aORs with 95% CIs for adherence (yes = 1) vs. non-adherence (no = 0) to the algorithm. The covariates encompassed age ≥65 years, initial severity grade, treatment status (primary vs. secondary), facility type (clinic [reference], affiliated hospital, university hospital), comorbidities (diabetes, hypertension, dyslipidemia), and days from onset to start of initial therapy (0–3 [reference], 4–7, 8–14, ≥15). Estimates were obtained using logistic regression with robust standard errors. Model discrimination: AUC = 0.86; maximum VIF = 3.56. Values >1 indicate higher odds of adherence; values < 1 indicate lower odds of adherence. Full numerical results are provided in Table 3 of the main text. aOR, adjusted odds ratio; AUC, area under the curve; CI, confidence interval; VIF, variance inflation factor. [file Image_2.tiff]

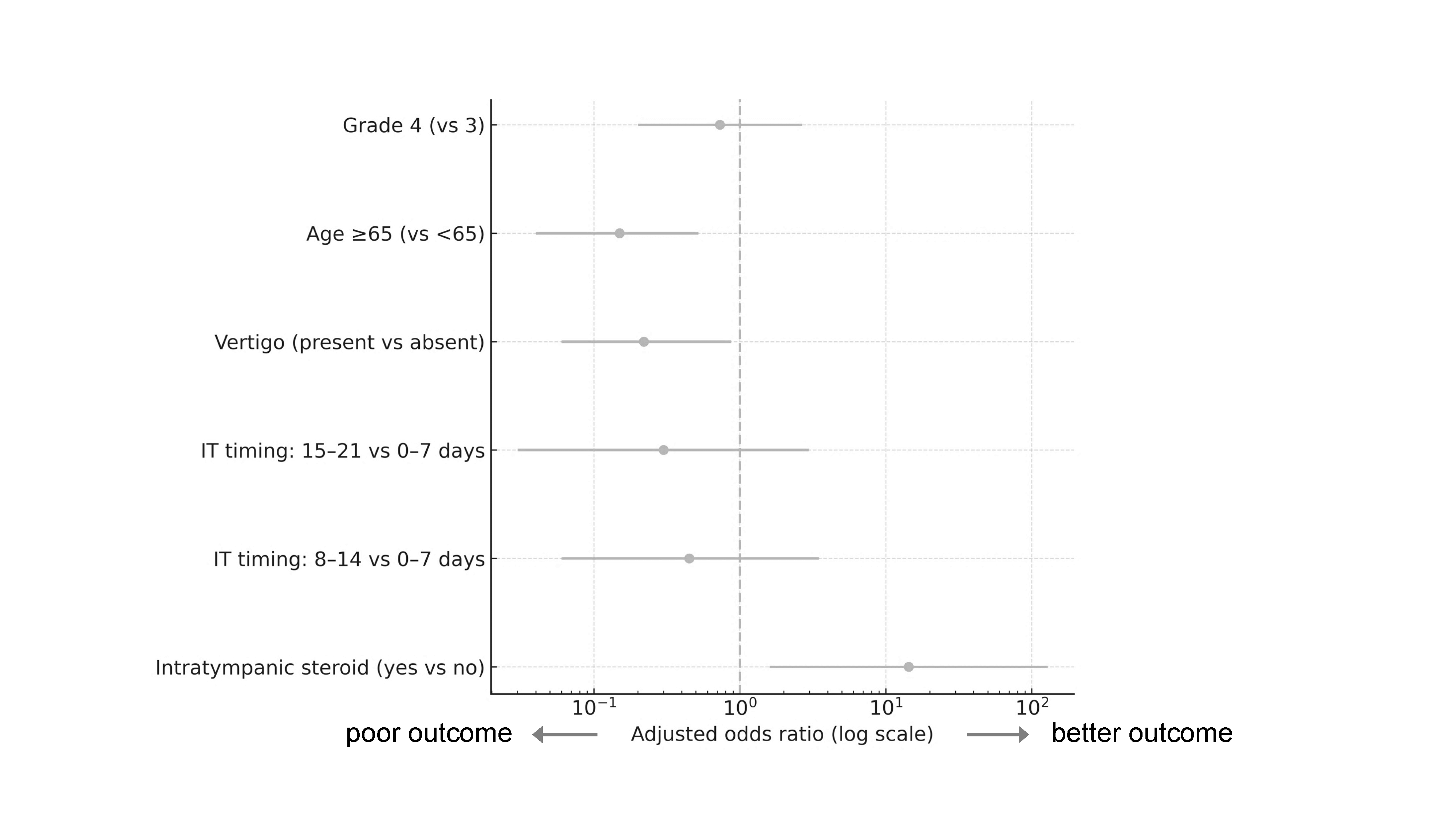

Supplement: Supplementary Figure 3 — Determinants of “marked improvement or better” among salvage-eligible severe SSNHL cases (multivariable logistic regression; forest plot). Among Grade ≥3/4 patients who were salvage-eligible after initial therapy ( ≤ 30 dB improvement at completion of initial therapy), this forest plot shows aORs with 95% CIs for achieving “marked improvement or better” (≥30 dB improvement) at the fixed hearing assessment. The covariates included IT steroids (yes vs. no), timing of IT (0–7 days [reference], 8–14, 15–21 days from onset; modeled as an interaction with IT), initial severity (Grade 4 vs. 3), vertigo (present vs. absent), age ≥65 years, and treatment status (secondary vs. primary). Logistic models used robust standard errors; model AUC = 0.879. In the main text (Table 4), IT steroids were independently associated with better outcomes (aOR 14.35; 95% CI 1.60–128.42; p = 0.017), whereas vertigo and age ≥65 years were associated with poorer outcomes. aOR, adjusted odds ratio; AUC, area under the curve; CI, confidence interval; IT, intratympanic; SSNHL, sudden sensorineural hearing loss. [file Image_3.tiff]
