# Supplementary material for: Clinical impact of adherence to a standardized treatment algorithm for idiopathic sudden sensorineural hearing loss: a multicenter cohort study
Source: Front Neurol. 2026 Feb 18;17:1775755. doi: 10.3389/fneur.2026.1775755 (PMC12956656; doi:10.3389/fneur.2026.1775755)
Supplement: Table S1 — List of participating facilities in the multicenter study. [file Table_1.docx]

**Supplementary Table S1. Participating facilities.**

| **No.** | **English name** |
| --- | --- |
| 1 | Kobayashi Otorhinolaryngology Clinic |
| 2 | OGUCHI E.N.T. Clinic |
| 3 | Iiyama Red Cross Hospital |
| 4 | Iizuna Municipal Hospital |
| 5 | Nagano Chuo Hospital |
| 6 | South Nagano Medical Center Shinmachi Hospital |
| 7 | Karuizawa Municipal Hospital |
| 8 | Asama Nanroku Komoro Medical Center |
| 9 | JA Nagano Koseiren Kakeyu‑Misayama Rehabilitation Center Kakeyu Hospital |
| 10 | Maruko Central Hospital |
| 11 | Fujimi Kogen Hospital |
| 12 | Matsumoto City Hospital |
| 13 | Showa Inan General Hospital |
| 14 | North Alps Medical Center Azumi Hospital |
| 15 | Omachi Municipal General Hospital |
| 16 | Nagano Prefectural Kiso Hospital |
| 17 | Yodakubo Hospital |
| 18 | Azumino Red Cross Hospital |
