# Supplementary material for: Clinical impact of adherence to a standardized treatment algorithm for idiopathic sudden sensorineural hearing loss: a multicenter cohort study
Source: Front Neurol. 2026 Feb 18;17:1775755. doi: 10.3389/fneur.2026.1775755 (PMC12956656; doi:10.3389/fneur.2026.1775755)
Supplement: Table S2 — SSNHL diagnostic criteria, severity grading, and outcome criteria used in Japan: (A) diagnostic criteria for idiopathic SSNHL; (B) severity grading criteria; (C) hearing improvement criteria. [file Table_2.docx]

**Supplementary Table S2. Diagnostic criteria, hearing-loss grading, and recovery criteria for idiopathic sudden sensorineural hearing loss (iSSNHL) in Japan.**

Abbreviation: iSSNHL, idiopathic sudden sensorineural hearing loss.

**A) Diagnostic criteria of iSSNHL in Japan (Intractable Hearing Loss Research Committee of the Ministry of Health, Labour and Welfare, revised 2015).**

Main symptoms:

- Sudden onset
- Sensorineural hearing loss
- Unknown etiology

For reference:

- Hearing loss (i.e., hearing loss of 30 dB or more over three consecutive frequencies)
- Sudden onset of hearing loss, but may progressively deteriorate over 72 h
- No history of recurrent episodes
- May be accompanied by tinnitus
- May be accompanied by vertigo, nausea, and/or vomiting, without recurrent episodes
- No cranial nerve symptoms other than from cranial nerve VIII

Definite diagnosis: all of the above main symptoms are present.

**B) Criteria for the grading of hearing loss in iSSNHL (Intractable Hearing Loss Research Committee of the Ministry of Health, Labour and Welfare, revised 2015).**

5-frequency average hearing level in the affected ear at 250, 500, 1000, 2000, and 4000 Hz (dB HL).

| **Grade** | **Criteria** |
| --- | --- |
| Grade 1 | <40 |
| Grade 2 | 40 to <60 |
| Grade 3 | 60 to <90 |
| Grade 4 | ≥90 |

**C) Hearing recovery criteria (Intractable Hearing Loss Research Committee of the Ministry of Health, Labour and Welfare, revised 2015).**

| **Recovery group** | **Criteria** |
| --- | --- |
| Complete recovery | Final hearing ≤20 dB, or level equal to that of the contralateral ear |
| Marked recovery | Hearing gain ≥30 dB |
| Slight recovery | Hearing gain 10–29 dB |
| No recovery | Hearing gain <10 dB |

Better outcome was defined as complete recovery or marked recovery; poor outcome as slight recovery or no recovery.
